# I Didn’t Really Fully Understand Until I Came Into the States: African Immigrants’ ADRD Introduction and Awareness

**DOI:** 10.1093/geroni/igab046.1784

**Published:** 2021-12-17

**Authors:** Manka Nkimbeng, Kwame Akosah, Tetyana Shippee, Christina Rosebush, Wynfred Russell, Joseph Gaugler

**Affiliations:** 1 University of Minnesota, Minneapolis, Minnesota, United States; 2 University of Minnesota, University of Minnesota, Minnesota, United States; 3 University of Minnesota, Twin Cities, Minneapolis, Minnesota, United States; 4 The African, Career Resources and Education Inc, Brooklyn Park, Minnesota, United States

## Abstract

Most African immigrants report that they had never heard about dementia until their arrival in the United States. Conversations and insights from project advisory board meetings of the African Immigrant Memory Loss and Dementia Education projects (5 conversations and 8 meetings in the Minneapolis area) reveal unique cultural and immigrant characteristics surrounding dementia terminology and awareness. Dementia is often lumped together with mental illness which is associated with stigma. In addition to the fear of bad news and death, mental health issues are often considered a result of witchcraft, spiritual attack or punishment. Additionally, there are no traditional or cultural words for dementia in many African languages and current terms used are related to mental illness and all have negative connotations. There is a need to identify appropriate words for dementia in many tribal and immigrant dialects that can facilitate dementia awareness and education programs in African communities.

